# The complete mitochondrial genome of *Sarcophaga polystylata* (Diptera: Sarcophagidae)

**DOI:** 10.1080/23802359.2021.2022542

**Published:** 2022-01-18

**Authors:** Yaoqing Chen, Yuxin Wang, Xiangyan Zhang, Jianbo Li, Ying Zou, Yadong Guo

**Affiliations:** a Department of Criminal Science and Technology, Hunan Police Academy, Changsha, Hunan, China; b Xiangya School of Pharmaceutical Sciences, Central South University, Changsha, Hunan, China; c Department of Forensic Science, School of Basic Medical Sciences, Central South University, Changsha, Hunan, China; d Criminal Investigation Detachment of Public Security Bureau of Changsha, Changsha, Hunan, China

**Keywords:** Mitochondrial genome, *Sarcophaga polystylata*, phylogenetic analysis

## Abstract

*Sarcophaga polystylata* (Ho, 1934) remains to be of medical and epidemiological significance. Here, we report the complete mitochondrial genome (mitogenome) of *S. polystylata* (GenBank accession no. MW592361). The length of this mitogenome was 15,233 bp with 39.4% A, 9.5% G, 14.3% C, and 36.8% T. The mitogenome of *S. polystylata* was composed of 13 protein-coding genes (PCGs), two ribosomal RNAs (rRNAs), 22 transfer RNAs (tRNAs), and a non-coding control region. Phylogenetic tree indicated that *S. polystylata* and *Sarcophaga peregrina* clustered together closely, both of which separated clearly from other species. This study provides the mitochondrial genetic data of *S. polystylata* for further understanding the phylogenetic relationship of sarcophagids species.

Sarcophagid fly causes global health concerns as a carrier of infectious viruses. Besides the medical significance, sarcophagid fly also has forensic significance in forensic entomology (Ren et al. 2018). *Sarcophaga (Liosarcophaga) polystylata* (Ho, 1934) (Diptera: Sarcophagidae) is widely distributed in China (including Beijing, Hebei, Heilongjiang, Henan, Jiangsu, Jilin, Liaoning, Shaanxi, Shandong, Shanxi, Sichuan, Guangxi, Guizhou, and Zhejiang), Japan (including Hokkaido, Honshu, Kyushu, and Shikoku), Russia (including Far East and West Siberia), and South Korea (Pape 1996; Xu and Zhao 1996). Larvae of *S. polystylata* are polyphagous, and can feed on carrion as well as human feces (Xu and Zhao 1996). Here, we present the complete mitochondrial genome (mitogenome) of *S. polystylata*, which would further enrich our understanding of the phylogenetic relationship of *Liosarcophaga* subgenus (Cameron 2014).

Adult specimens of *S. polystylata* were trapped in Beijing (39°56′ N, 116°20′ E), China, in June 2020. All specimens were sacrificed by freezing methods, and then identified by traditional morphological methods (Xu and Zhao 1996). After that, the specimens were stored at −80 °C in Guo’s lab (Changsha, China) with a unique code (CSU20210204). Total genomic DNA was extracted from thoracic muscles of an adult specimen using QIANamp Micro DNA Kit (QIANGEN Biotech Co., Ltd., Beijing, China) following the manufacturer’s instruction. The mitogenome sequencing of *S. polystylata* was performed on an Illumina HiSeq 4000 Platform (pared-end 150 bp), and then assembled based on Illumina short reads with MitoZ v2.3 (Meng et al. 2019). The rough boundaries of all genes were annotated by MITOS2 Web Server based on invertebrate mitochondrial genetic code (Bernt et al. 2013).

In this study, total length of the mitogenome of *S. polystylata* was 15,233 bp (GenBank accession no. MW592361), containing 13 protein-coding genes (PCGs), two ribosomal RNAs (rRNAs), 22 transfer RNAs (tRNAs), and a non-coding control region. The gene arrangement is consistent with that of ancestral metazoan (Cameron 2014). All PCGs initiated with a typical start codon of ATN, except that cox1 started with TCG. Most PCGs terminated with TAA/TAG, in addition that four genes (*cox1*, *cox2*, *nad4*, and *nad5*) terminated with T. Nucleotide composition of S. *polystylata* was 39.4% A, 9.5% G, 14.3% C, and 36.8% T, indicating a highly A + T bias (76.2%). In addition, the size of overlap regions was examined, varying from 1 to 9 base pairs. Furthermore, the size of intergenic spacers varied from 1 to 21 base pairs. The longest intergenic spacers (21 bp) were situated between tRNA-GLU and tRNA-PHE.

Moreover, phylogenetic analysis of *S. polystylata* with nine sarcophagids species was constructed using maximum-likelihood (ML) method based on the 13 PCGs, and *Calliphora vomitoria* (Diptera: Calliphoridae) was used as an outgroup (Figure 1). ML analysis was performed with the GTR + G+I model implemented in IQ-TREE v.1.6.12 (Nguyen et al. 2015). In the phylogenetic tree, *S. polystylata* and *Sarcophaga peregrina* were closely clustered and clearly separated from other species. The branches were well supported. The clusters of *S. dux* and *S. angarosinica* emerged as sister to *S. polystylata* and *S. peregrina*. While the phylogenetic analysis also presented the sister-group between these four species and the remaining six species with high branch support. This study provides mitochondrial genetic data of *S. polystylata* for further studying on evolutionary relationship of sarcophagids species.

**Figure 1.**
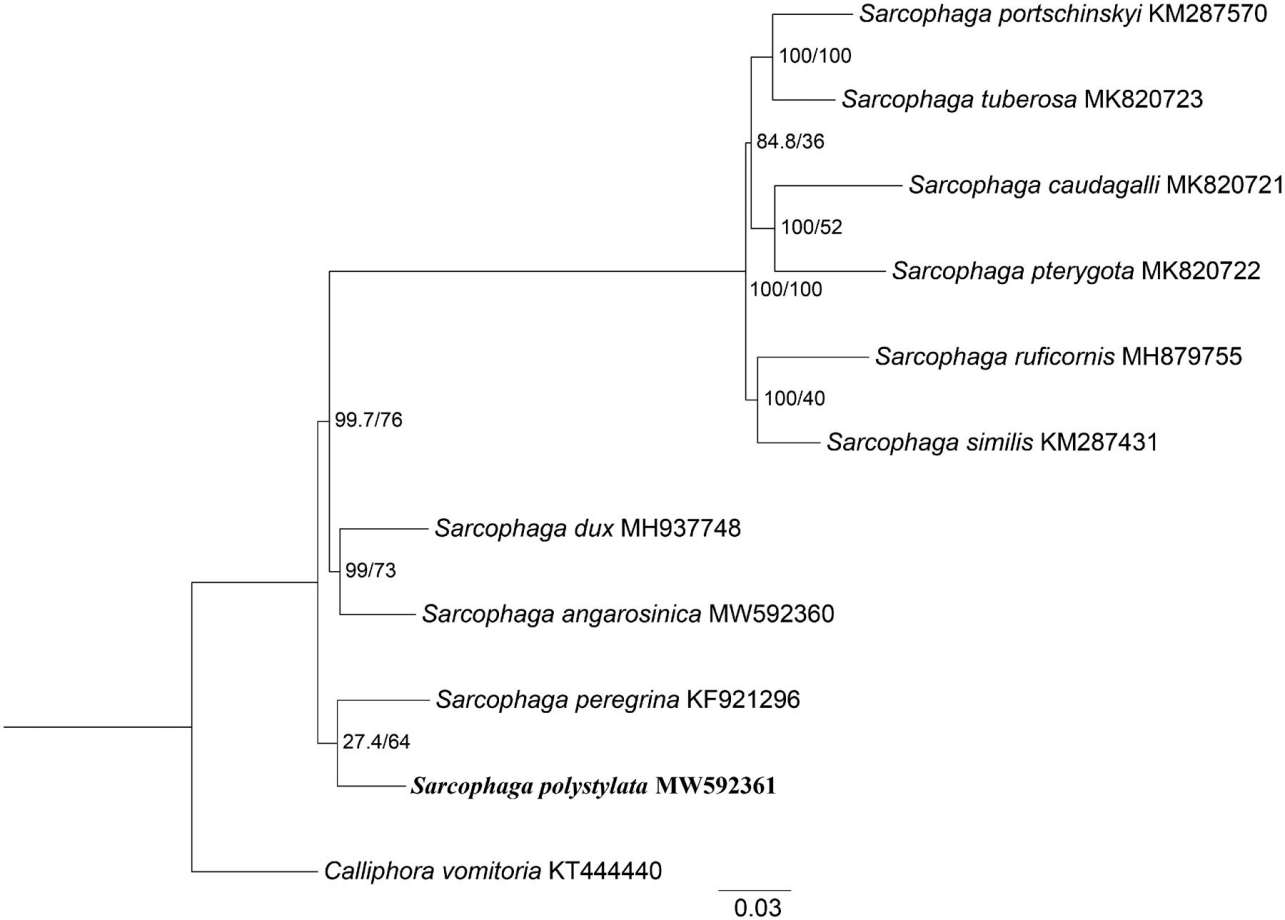
Phylogenetic trees of *S. polystylata* with nine sarcophagids species based on 13 PCGs by maximum likelihood (ML) method. *Calliphora vomitoria* was selected as an outgroup.

## Data Availability

The data that support the findings of this study are openly available in NCBI at https://www.ncbi.nlm.nih.gov (GenBank: MW592361, BioProject: PRJNA700703, BioSample: SAMN17838723, and SRA: SRR13757412).
